# Cuprizone markedly decreases kynurenic acid levels in the rodent brain tissue and plasma

**DOI:** 10.1016/j.heliyon.2021.e06124

**Published:** 2021-02-01

**Authors:** Helga Polyák, Edina Katalin Cseh, Zsuzsanna Bohár, Cecilia Rajda, Dénes Zádori, Péter Klivényi, József Toldi, László Vécsei

**Affiliations:** a Department of Neurology, Interdisciplinary Centre of Excellence, Faculty of Medicine, Albert Szent-Györgyi Clinical Centre, University of Szeged, Szeged, Hungary; b MTA-SZTE Neuroscience Research Group, Szeged, Hungary; c Department of Physiology, Anatomy and Neuroscience, University of Szeged, Szeged, Hungary

**Keywords:** Kynurenine pathway, Kynurenic acid, Cuprizone, Multiple sclerosis, Demyelination, Remyelination

## Abstract

**Background:**

The kynurenine (KYN) pathway (KP) of the tryptophan (TRP) metabolism seems to play a role in the pathomechanism of multiple sclerosis (MS). Cuprizone (CPZ) treated animals develop both demyelination (DEM) and remyelination (REM) in lack of peripheral immune response, such as the lesion pattern type III and IV in MS, representing primary oligodendrogliopathy.

**Objective:**

To measure the metabolites of the KP in the CPZ treated animals, including TRP, KYN and kynurenic acid (KYNA). We proposed that KYNA levels might be decreased in the CPZ-induced demyelinating phase of the animal model of MS, which model represents the progressive phase of the disease.

**Methods:**

A total of 64 C57Bl/6J animals were used for the study. Immunohistochemical (IHC) measurements were performed to prove the effect of CPZ, whereas high-performance liquid chromatography (HPLC) was used to quantify the metabolites of the KP (n = 10/4 groups; DEM, CO1, REM, CO2).

**Results:**

IHC measurements proved the detrimental effects of CPZ. HPLC measurements demonstrated a decrease of KYNA in the hippocampus (p < 0.05), somatosensory cortex (p < 0.01) and in plasma (p < 0.001).

**Conclusion:**

This is the first evidence of marked reduction in KYNA levels in a non-immune mediated model of MS. Our results suggest an involvement of the KP in the pathomechanism of MS, which needs to be further elucidated.

## 1 Introduction

Multiple sclerosis (MS) is an immune mediated, chronic inflammatory and demyelinating disease of the central nervous system (CNS). According to a recent study (Browne et al., 2014), roughly 2.3 million people world-wide are affected by this disorder. It starts in young adulthood and it has a great impact on the quality of life. The onset is characterized by demyelination (DEM), lesions with loss of axons and degeneration of neurons (Lassmann and Bradl, 2017). Although the precise pathophysiology still remains to be clarified, oxidative stress is known to play a major role in the development of the disease (Rajda et al., 2017). Beside this, blood brain barrier (BBB) disruption and immune system dysregulation (Høglund and Maghazachi, 2014), pro- and anti-inflammatory cytokine disequilibrium (Trenova and Slavov, 2015) and activation of glial cells (Ponath et al., 2018) were described to be important too. As in the animal models of MS, in the human disease, mature oligodendrocytes (OLGs) are already affected at the onset of the disease. Microglia and macrophage activation occur to purify myelin debris, thereby retain the myelin sheath via remyelination. Over time, the remyelination efficiency is impaired due to the overloading of the pathophysiological processes of demyelination. The infiltration of CNS by peripheral immune cells (T and/or B cells) and macrophages, as well as the activation of microglia and astrocytes within the CNS, result in the exaggerated production of reactive oxygen species (ROS) and proinflammatory cytokines (Ortiz et al., 2016). Excessive ROS production and reduced antioxidant activity leads to oxidative stress (Adamczyk and Adamczyk-Sowa, 2016).

For the investigation and a better understanding of this disease, several animal models are used, and one of them is the bis(cyclohexanone)oxaldihydrazone (Cuprizone, CPZ)-induced demyelination model of MS (for review, see (Sen et al., 2019)).

The CPZ model aims to investigate the mechanisms of demyelination and remyelination in lack of peripheral immune response (Sen et al., 2019). CPZ influences the function of the mitochondrial respiratory chain by the inhibition of mitochondrial complex IV, as well as complex I-III and complex II-III (for review, see (Praet et al., 2014)). The major histological appearance of CPZ-induced lesions is largely comparable to the type III and IV lesion pattern in MS, which are characterized by demyelination, oligodendroglial cell death and microglia/macrophage activation (Sen et al., 2019). They are associated with increased levels of ROS and reactive nitrogen species (Wakabayashi, 2002), while the BBB remains intact (Sen et al., 2019). CPZ intoxication-induced mitochondrial dysfunction contributes to the loss of OLGs and axons (Kalman et al., 2007). In early stages of the CPZ treatment the selective apoptotic death of mature OLGs was described (Mason et al., 2001). This feature was accompanied by oligodendroglial cell death not equally distributed in the CNS, extended demyelination and oligodendroglial cell death occurring in the cerebellum and the different cerebrum areas, such as corpus callosum, cerebral cortex, hippocampus or striatum, but the brainstem and spinal cord are also affected to a limited extent (Sen et al., 2019). Remyelination starts with the accumulation of oligodendrocyte progenitor cells (OPC) following 3 weeks of CPZ intoxication with astrocyte and microglia activation and it becomes evident by week 6 of CPZ treatment, when OPCs become mature, myelinating OLGs (Praet et al., 2014).

**Figure 1:**
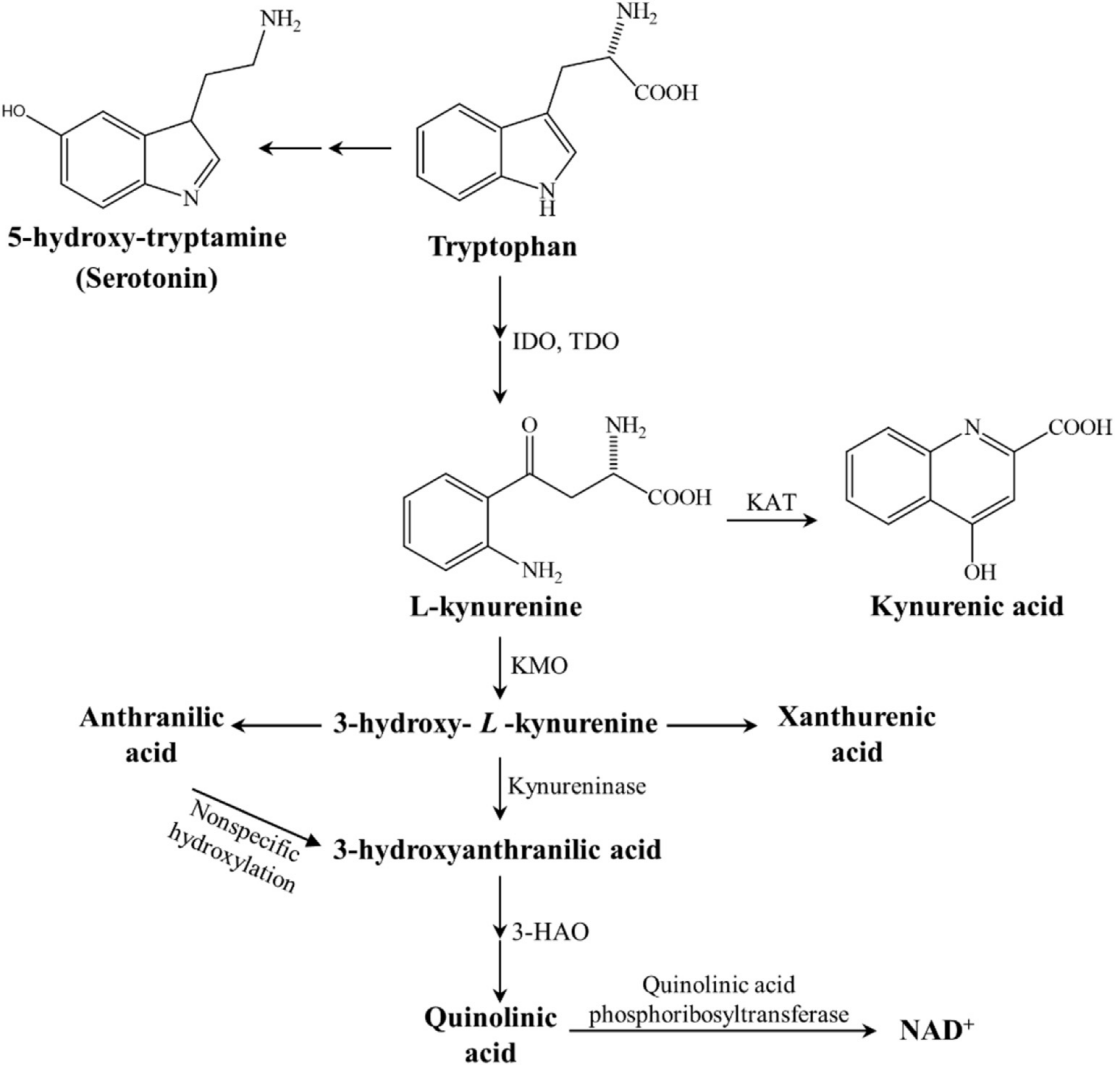
The kynurenine metabolite pathway. *3-HAO* 3-hydroxyanthranilate oxidase, *IDO/TDO* indoleamine 2,3-dioxygenase/tryptophan 2,3-dioxygenase, *KAT* kynurenine aminotransferase, *KMO* kynurenine 3-monooxygenase*, NAD*^*+*^ nicotineamide adenine dinucleotide.

Kynurenic acid (KYNA) is an endogenous N-methyl-D-aspartate (NMDA) receptor antagonist, which is produced by the kynurenine (KYN) pathway (KP) (Figure 1), whereas KYN is an intermediate metabolite of the KP, which can be metabolized via three different pathways to form KYNA, xanthurenic acid or nicotinamide adenine dinucleotide (NAD+) (Vécsei et al., 2013). KYNA is produced by KYN aminotransferases (KATs) and it can influence the glutamatergic transmission in different ways (Zádori et al., 2011), i.e., it behaves as a competitive antagonist on the NMDA receptor (Kessler et al., 1989) and has weak antagonistic effects on the AMPA- and kainate receptors (Birch et al., 1988). Furthermore, KYNA is a potent endogenous aryl hydrocarbon-receptor ligand, suggesting a significant immunomodulatory role too (Bohár et al., 2015). The KP shows alterations in different neurological disorders, including MS (for review, see (Vécsei et al., 2013; Zádori et al., 2011, 2009). While in relapsing MS the KP is shifted towards the neurotoxic metabolite quinolinic acid (QUIN), the progressing form is characterized by decreased KYNA levels (Lim et al., 2017; Lovelace et al., 2016).

Based on the above mentioned data, the aim of the current study was to measure the metabolites of the KP in CPZ treated animals, including TRP, KYN and KYNA. We proposed that KYNA levels might be decreased in the CPZ-induced demyelinating phase of the animal model of MS, as this the best model, which represents the progressive phase of the disease.

## 2 Materials and methods

### 2.1 Animal experiments and sample collection

Eight weeks old (20–25 g) male C57Bl/6J mice were used (n = 64). The animals were bred and maintained under standard laboratory conditions with 12 h–12 h light/dark cycle at 24 ± 1 °C and 45–55% relative humidity in the Animal House of the Department of Neurology, University of Szeged. The studies were in accordance with the Ethical Codex of Animal Experiments and were approved by the Committee of the Animal Research of University of Szeged (I-74-49/2017) and were authorized by the National Food Chain Safety Office with a permission number of XI/1101/2018, as well as followed the guidelines the Use of Animals in Research of the International Association for the Study of Pain and the directive of the European Parliament (2010/63/EU). The animals were housed in groups of 4–5 in polycarbonate cages (530 cm^3^ floor space). Prior to the experiment, the animals were habituated to grounded standard rodent chow, and they were measured every other day.

**Figure 2:**
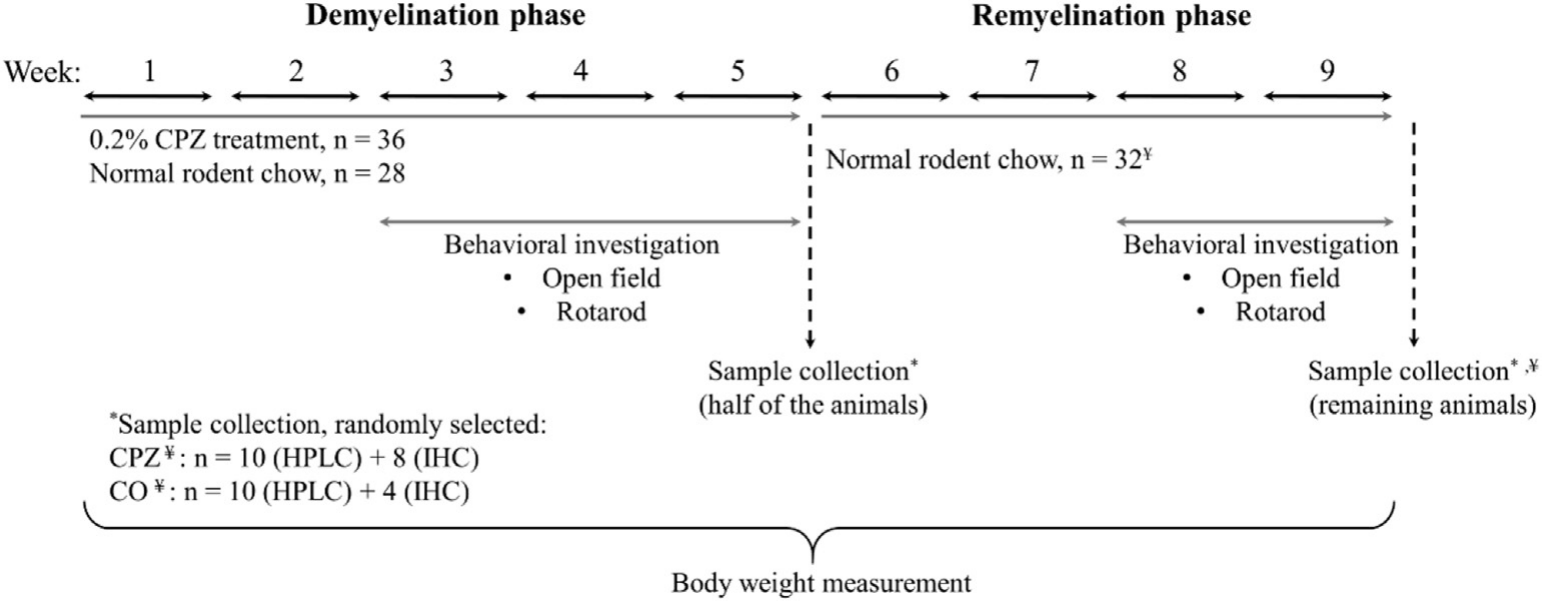
Timeline of the experimental procedure applied in this study. *CPZ* cuprizone; *CO* control; *IHC* immunohistochemical studies; *HPLC* High-performance liquid chromatography; ^¥^One animal died in cage at the beginning of the remyelination phase in the CPZ treated group and at the end of the remyelination phase, an animal also died in the CO group before perfusion. n represents the number of animals used in one group. ∗Sample collection was randomly selected in the CPZ treated- and the CO group.

DEM was induced by feeding mice a diet containing 0.2% CPZ (bis-cyclohexanone–oxaldihydrazone; Sigma-Aldrich, n = 36) mixed into a grounded standard rodent chow for 5 weeks. Age matched animals were used as controls (n = 28), which also had free access to grounded standard rodent chow and water. At the end of the five-week treatment period, in both cuprizone (CPZ) treated (n = 36) and control (CO) group (n = 28) half of the animals (CPZ DEM: n = 18, CO n = 14) were randomly chosen and sacrificed, as detailed below. At the end of 5-weeks of treatment, after perfusion, the cuprizone diet was excluded and from the 6th week onwards, the surviving animals participated in the remyelination (REM) phase for 4 weeks. At the end of the last week, the remaining animals were sacrificed (CPZ REM: n = 18, CO n = 14) as detailed below. Behavioral tests were performed during both the DEM and REM phases of the experiment (Figure 2). During the experiment, two animals died: one died in cage at the beginning of the remyelination phase in the CPZ treated group, presumably due to the change of the hierarchy between males, whereas the second one from the CO group died at the end of the remyelination phase, during anesthesia, right before perfusion.

The mice were anesthetized with intraperitoneal 4% chloral hydrate (10 ml/kg body weight). For the histological and immunohistochemical (IHC) studies mice (CPZ: n = 16, CO: n = 8) were perfused transcardially with artificial cerebrospinal fluid followed by 4% paraformaldehyde in 0.1 M phosphate buffer. Brain samples were dissected and postfixed in the same fixative overnight at 4 °C. Brains were embedded in paraffin, coronally sectioned in 8 μm thickness obtained from different regions (0.14, −0.22, −1.06, and −1.94 mm) according to the mouse brain atlas of Paxinos and Franklin (2001) and placed on gelatin-coated slides (Acs et al., 2009). For bioanalytical measurements, the animals (CPZ: n = 20, CO: n = 20) were anesthetized and perfused as described above. Blood samples were taken from the left heart ventricle into Eppendorf tubes containing disodium ethylenediaminetetraacetate dihydrate and the plasma was separated by centrifugation (3500 rpm for 10 min at 4 °C). The brains were dissected into five different brain regions, including the cerebellum, brainstem, striatum, somatosensory cortex and hippocampus. All samples were removed on ice and stored at -80 °C until further use. The treated and recovered mice were marked as groups DEM and REM, respectively, whereas age matched animals as controls became the CO1- and CO2 group.

### 2.2 Luxol Fast Blue (LFB) staining and myelin status analysis

Myelin damage was evaluated with Luxol Fast Blue-Cresyl-Violet (LFB/CV) staining. The brain slides were deparaffinized, rehydrated with 95% alcohol and incubated in a 0.01% LFB solution overnight at 60 °C, after that the sections were differentiated in 0.05% lithium carbonate solution and counterstained with CV.

### 2.3 Immunohistochemical and intensity measurement analysis

For IHC analysis gelatin-coated slides were deparaffinized, rehydrated and heat-unmasked in 10 mM citrate buffer. Sections were blocked with 0.3% hydrogen peroxide in phosphate-buffered saline (PBS) solution and incubated with the primary antibody diluted in a solution containing 0.1 M PBS and 10% normal goat or horse serum overnight. Anti-glial fibrillary acidic protein (GFAP, 1:500, rabbit IgG, Dako, Agilent) for astrocyte visualization and anti-myelin basic protein (MBP, 1:500, mouse IgG, Abcam) to detect myelin were applied. Adequate biotinylated secondary antibody was used and the ABC Kit (Vectastain Kit, Vector Laboratories) and 3,3′-diaminobenzidine (DAB) reaction was used for visualization. For measurements an AxioImager M2 microscope, equipped with an AxioCam MRc was used. Rev 3 camera (Carl Zeiss Microscopy) and AxioVision 4.8 software (Carl Zeiss Microscopy, Germany) program were applied. Quantification of astrogliosis was achieved by manual cell counting of the GFAP-immunopositive cells in the corpus callosum (CC). Intensity measurement was used to determine myelin content with MBP and LFB staining.

### 2.4 High-performance liquid chromatography (HPLC) measurement

Chromatographic separation was carried out with a validated method, as described before (Cseh et al., 2019). Briefly, on the day of measurement, plasma and brain samples were deproteinized by precipitation as described before (Cseh et al., 2019). The mobile phase, in each case was a 200 mM zinc acetate solution, at final pH of 6.2 for plasma, and 5.8 for brain tissue samples, with a final concentration of 5 % of acetonitrile. Regarding the LOD values, in brain samples it was 0.259 nmol/g ww for KYN and 1.82 for KYNA, whereas in plasma samples it was 1.33 nM for KYNA.

### 2.5 Behavioral investigation

During the open field test, each animal (n = 18/group) in CPZ treated and CO groups was placed in the center of an open-field box measuring 48 ∗ 48 ∗ 40 cm for 15 min tracking periods (analyzed in three 5 min periods). The movement patterns of the animals were tracked and recorded with the Conducta 1.0 system (Experimetria Ltd.). The parameters recorded were the ambulation distance, the time spent in immobility and the total time spent with consecutive rearing (Veres et al., 2015). Open field measurements were carried out in the third, fourth and fifth week of CPZ treatment and in the third and fourth week of the remyelination phase, once a week on the same day, in the morning.

Rotarod test was used to determine the effects of CPZ on motor function. The animals in the CPZ and CO groups (n = 18 animals/group) were trained on the rotarod for a 3-session period for 5 min on 2 consecutive days prior to the first test day. On the first and second days of the training sessions, a constant speed of 5 and 10 rpm respectively, was used. The test was performed on the third, fourth and fifth week of CPZ treatment and on the third and fourth week of the remyelination phase, once a week on the same day following the training sessions. The performance of each mouse was measured three times with resting periods of 30 min between consecutive tracking sessions. The latencies to fall values were recorded with the TSE Rotarod Advanced system. In the test phase, rodents were placed on a rotating rod, with a constantly increasing speed from 5 to 40 rpm during the 300 min test period. On the day before the respective test day, a 3 session retraining at 10 rpm for 5 min with 30 min resting periods was carried out to enable the animals to recall the rotarod experience (Veres et al., 2015).

### 2.6 Statistical analysis

For the quantitative analysis of GFAP-immunopositive cells and the analysis of intensity measurement of LFB and MBP statistical differences were determined by one-way analysis of variance (ANOVA) followed by the Sidak or Tamhane's T2 post hoc test depending on variances of data, with p < 0.05 taken as statistically significant. For statistical comparison of the body weight and behavioral measurements, we used two-way repeated-measures ANOVA. Pairwise comparisons of group means were based on the estimated marginal means with Sidak or Tamhane's T2 post hoc test with adjustment for multiple comparisons. Group values are reported as means ± SEM, analyses were performed in SPSS Statistics software (version 20.0 for Windows, SPSS Inc). Regarding the HPLC measurements, if the distribution (Shapiro–Wilk test) was proven to be Gaussian and the variances were equal (Levene test) ANOVA was applied with Tukey HSD post hoc test for pairwise comparison, otherwise Kruskal-Wallis, with the Wilcoxon post hoc test was used. Data were plotted as median (1^st^‒3^rd^ quartile).

## 3 Results

### 3.1 Body weight

**Figure 3:**
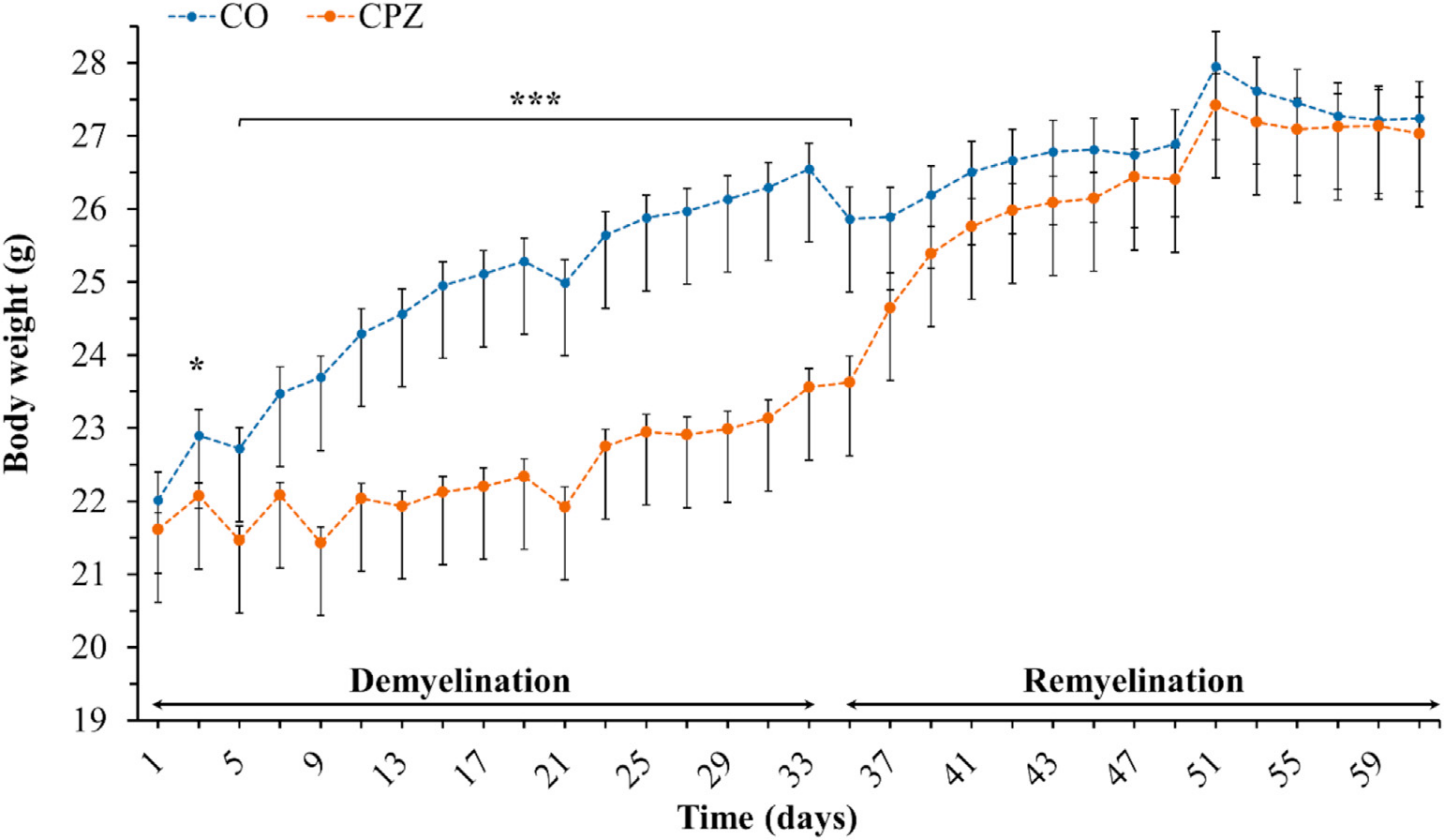
Changes in body weight of the animals during observation. *CO* controls, depicted with blue dots; *CPZ* cuprizone treated animals are highlighted with orange dots within two periods (demyelination- and remyelination phase) indicated with black arrows. ∗p < 0.05 *vs*. CO, ∗∗∗p < 0.001 *vs*. CO.

On the third day of CPZ treatment, there was already a significant difference in the body weight of the CPZ group compared to the CO group (Figure 3). This difference remained until the beginning of the REM phase. However, this difference disappeared during the REM phase and the body weight of the two groups returned to the same range by the end of the experiment.

### 3.2 Determination of CPZ damage after DEM and REM phase

**Figure 4:**
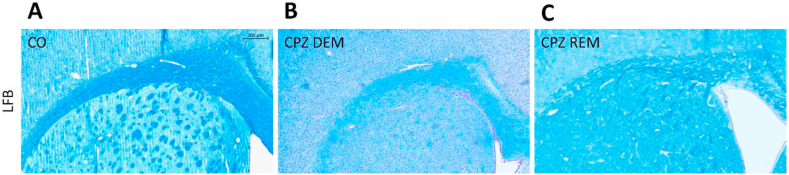
LFB staining in the corpus callosum of the CO- (A), DEM- (B) and REM (C) group. The CPZ-treated group (B) showed a reduced myelin content compared to the CO group (A), which increased during the remyelination phase (C). Scale bar: 200 μm. *CO* control group, *CPZ* cuprizone treated group, *DEM* demyelination phase in the treated group, *LFB* Luxol Fast Blue, *REM* remyelination phase in the treated group.

**Figure 5:**
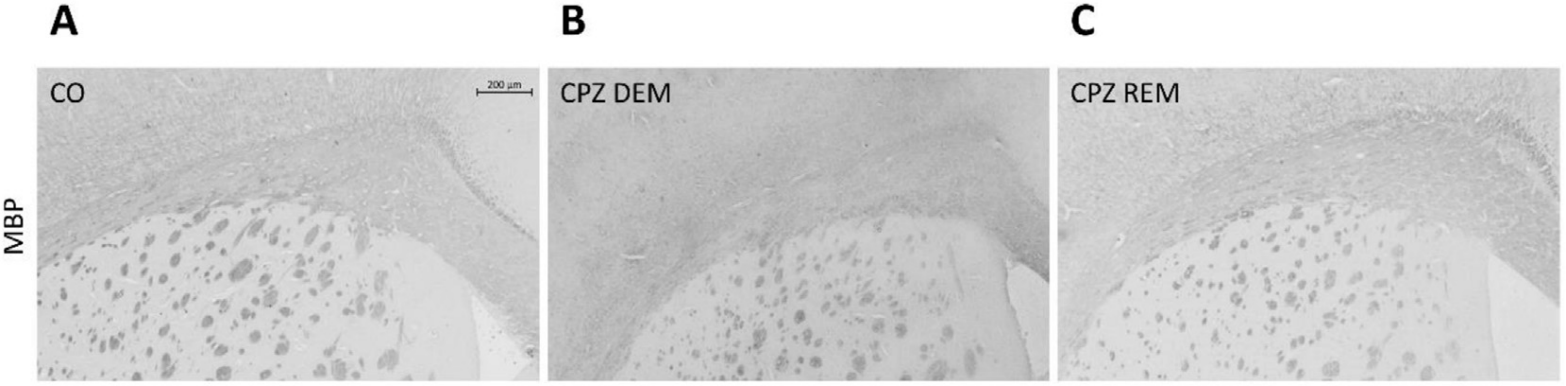
Myelin content determination of the corpus callosum with MBP staining in the CO (A), DEM (B) and REM (C) group, where the CPZ demyelination group (B) showed less myelin content, which increased in the remyelination phase (C). Scale bar: 200 μm. *CO* control group, *CPZ* cuprizone treated group, *DEM* demyelination phase of the treated group, *MBP* myelin basic protein, *REM* remyelination phase of the treated group.

**Figure 6:**
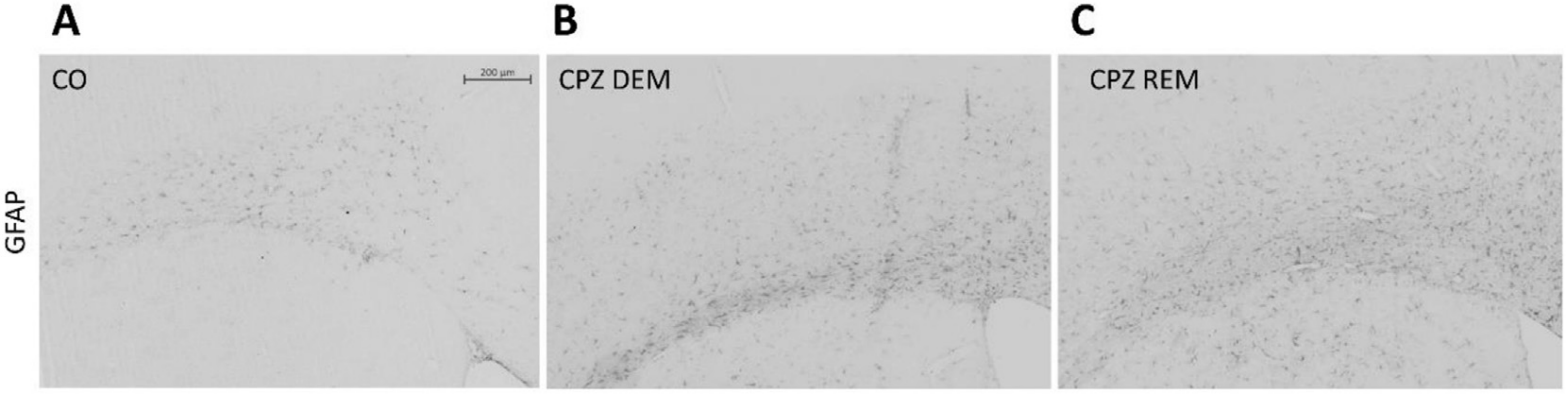
GFAP immunostaining in the corpus callosum of the CO- (A), DEM- (B) and REM (C) group. In the CO group (A) only a few astrocytes were present in the CC. During CPZ treatment an extensive astrogliosis was observed in the CPZ group (B), which persisted during remyelination phase (C). Scale bar: 200 μm. *CO* control group, *CPZ* cuprizone, *DEM* demyelination group, *GFAP* glial fibrillary acidic protein, *REM* remyelination group.

**Figure 7:**
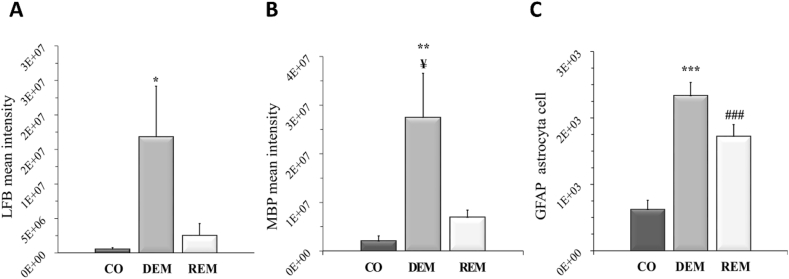
Effect of CPZ administration on myelination of the CC in the CO-, DEM- and REM group. LFB/CV (A) and MBP (B) staining for myelin content determination by intensity measurement. Immunohistochemical staining for GFAP (C) in the CC. Quantification of astrogliosis was done by manual cell counting. Our results show that the CPZ treatment caused a significant decrease in myelin content and an extensive astrogliosis in the CPZ group compared to the CO group. *CC* corpus callosum, *CO* control group, *DEM* demyelination group, *GFAP* glial fibrillary acidic protein, *LFB* Luxol Fast Blue, *MBP* myelin basic protein, *REM* remyelination group, ∗p < 0.05 *vs*. CO, ∗∗p < 0.01 *vs*. CO, ¥p < 0.05 *vs*. REM, ∗∗∗p < 0.001 *vs*. CO, ###p < 0.001 *vs*. CO.

Five weeks of CPZ feeding (acute DEM phase) resulted in an extensive and significant demyelination in the CC, as shown by LFB/CV (Figure 4) and MBP staining (Figure 5, Figure 7). This myelin damage was ameliorated at week four after REM. Immunostaining with GFAP cells (Figure 6) within the CC showed an extensive astrogliosis in the CPZ treated groups (DEM and REM) compared to the CO group (Figure 7).

### 3.3 Behavioral tests

**Figure 8:**
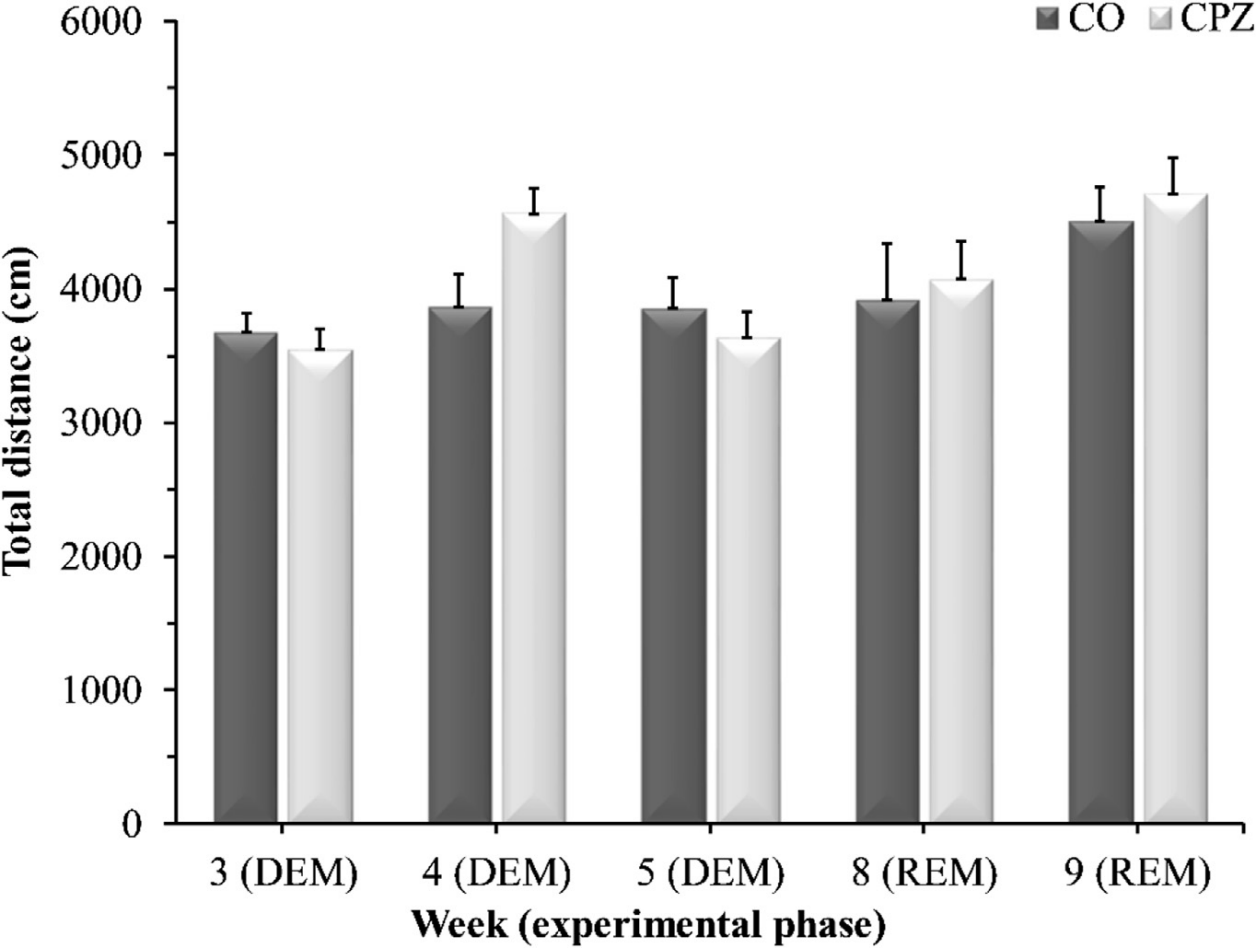
Open field test results. Data are presented as the total ambulation distance in the CO-, DEM- and REM groups. *CO* control group, *CPZ* cuprizone treated group, *DEM* demyelination phase in the treated group, *REM* remyelination phase in the treated group, n = 18/group.

**Figure 9:**
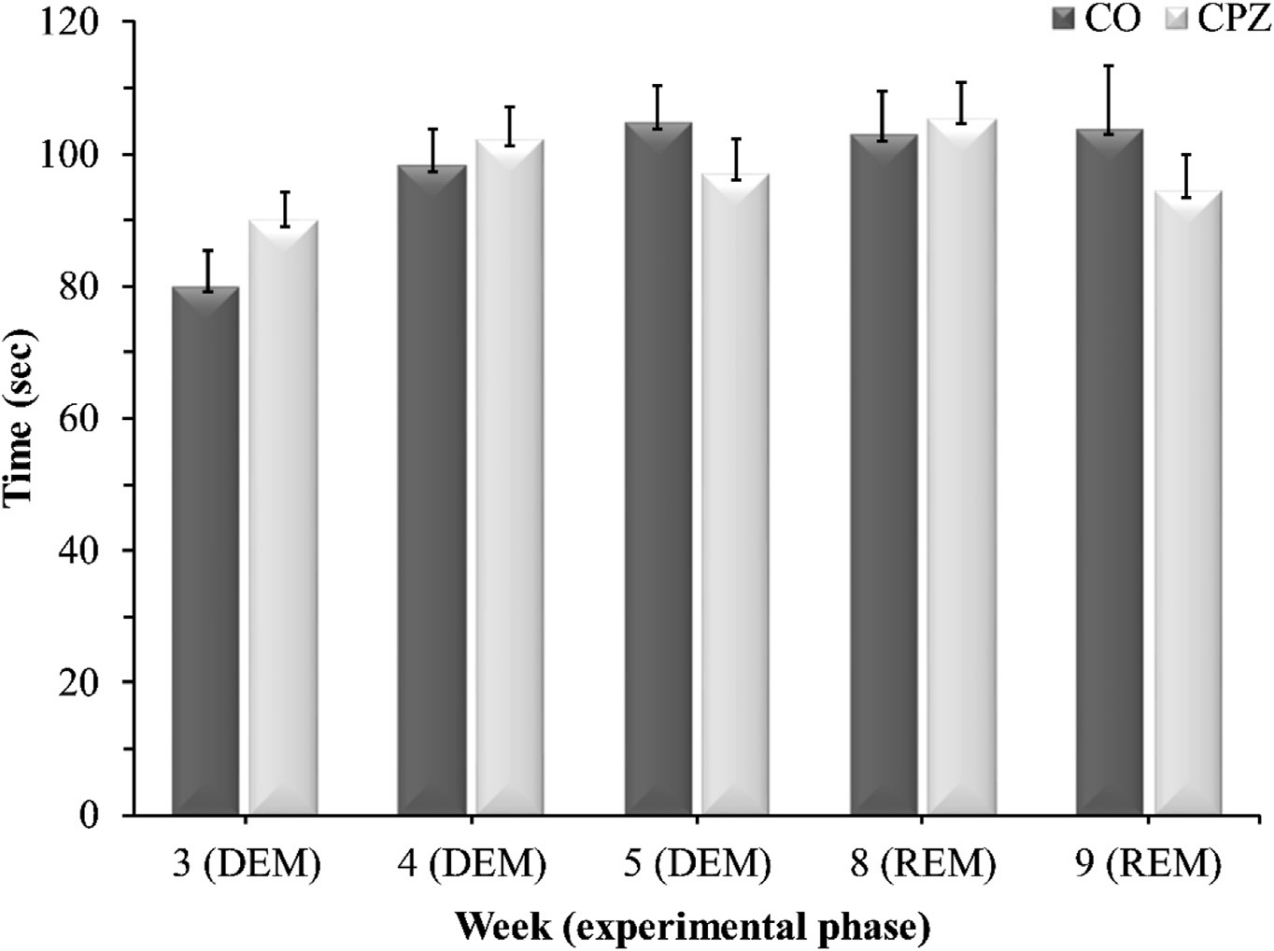
Rotarod investigation results. Data are presented as the total time spent on the rod in the CO-, DEM- and REM groups. *CO* control group, *CPZ* cuprizone treated group, *DEM* demyelination phase of the treated group, *REM* remyelination phase of the treated group, n = 18/group.

There were no significant differences between the CPZ- and CO groups during the DEM- or REM phase in any of the measured parameters in either the open field test (Figure 8), or in the rotarod test (Figure 9).

### 3.4 HPLC measurement of tryptophan metabolites from brain and plasma samples

**Figure 10:**
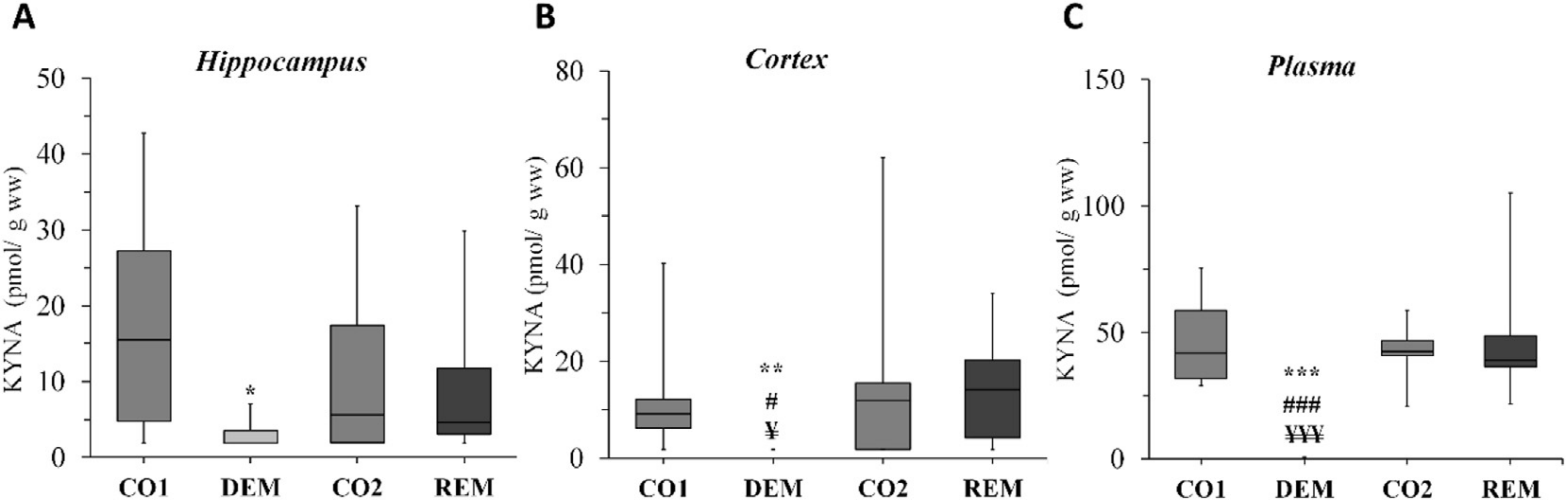
Significant decrease of kynurenic acid concentration in the cuprizone treated group (*demyelination group*)*.* n = 10/group, except DEM, where one animal died in cage after anesthesia. *CO1 – control group belonging to the cuprizone treated group (DEM) in the demyelination phase; CO2 – control group in the remyelination phase, DEM* demyelination phase of the treated group, *KYNA* kynurenic acid, *REM* remyelination phase of the treated group, *ww* wet weight. ∗p < 0.05 *vs.* CO1, ∗∗p < 0.01 *vs.* CO1, ∗∗∗p < 0.001 *vs.* CO1, ^#^p < 0.05 *vs.* CO2, ^###^p < 0.001 *vs.* CO2, ^¥^p < 0.05 *vs.* REM, ^¥¥¥^p < 0.001 *vs.* REM.

The mentioned five brain regions, i.e., cerebellum, brainstem, striatum, somatosensory cortex and hippocampus were analyzed during the bioanalytical measurements. All measured concentrations are presented in Table 1 including the ones from plasma samples. The boxplots represent only the significant changes observed after the sample measurements, namely the significant decrease of KYNA in the hippocampus (Figure 10A), cortex (Figure 10B) and plasma (Figure 10C). As the data distribution was not normal (p > 0.05), data were presented as mean (1^st^ and 3^rd^ quartile range). The other three metabolites (TRP, KYN and serotonin (5-HT)) did not change significantly in the assessed brain regions.

**Table 1:** Concentration levels of tryptophan, kynurenine, kynurenic acid and serotonin.

	CO1 (n = 10)	DEM (n = 10)	CO2 (n = 9†)	REM (n = 10)
*Hippocampus*				
TRP (nmol/g ww)	14.7 (13.9–19.9)	17.3 (15.6–18.3)	16.21 (13.1–16.9)	15.8 (14.8–18.1)
KYN (nmol/g ww)	<LOD	<LOD	<LOD	<LOD
KYNA (pmol/g ww)	15.5 (4.75–27.1)	<LOD∗ (<LOD ‒3.52)	5.61 (1.94–17.4)	4.61 (3.06–11.8)
5-HT (pmol/g ww)	3777 (3415–3978)	3813 (3538–3996)	4096 (3720–4119)	3824 (3607–4095)
*Somatosensory Cortex*				
TRP (nmol/g ww)	18.1 (16.4–22.2)	18.9 (17.9–20.6)	28.9 (25.2–30.6)	14.2 (4.25–20.2)
KYN (nmol/g ww)	<LOD	<LOD	<LOD	<LOD
KYNA (pmol/g ww)	10.7 (6.20–12.1)	<LOD∗∗^**,#,¥**^	11.9 (<LOD ‒15.6)	14.2 (4.25–20.2)
5-HT (pmol/g ww)	3916 (3477–4364)	4064 (3597–4317)	3322 (3208–3777)	3442 (2640–3969)
*Striatum*				
TRP (nmol/g ww)	20.6 (18.0–23.6)	18.7 (16.9–20.2)	17.1 (16.2–18.8)	17.1 (14.8–19.2)
KYN (nmol/g ww)	<LOD	<LOD	<LOD	<LOD
KYNA (pmol/g ww)	10.7 (7.19–22.1)	<LOD (<LOD‒3.66)	3.82 (<LOD‒10.0)	10.5 (6.42–20.3)
5-HT (pmol/g ww)	3943 (3654–4218)	3958 (3254–4453)	3831 (3580–4476)	3765 (3663–4236)
*Cerebellum*				
TRP (nmol/g ww)	18.0 (16.0–34.5)	19.6 (17.0–21.1)	16.9 (13.5–20.0)	18.3 (15.5–22.4)
KYN (nmol/g ww)	0.367 (<LOD‒0.439)	<LOD	<LOD	<LOD
KYNA (pmol/g ww)	7.3 (5.25–12.0)	<LOD	12.0 (3.05–17.7)	4.70 (2.25–7.89)
5-HT (pmol/g ww)	1734 (1533–2026)	1490 (1411–1962)	1477 (1066–3216)	1639 (1307–2453)
*Brainstem*				
TRP (nmol/g ww)	14.9 (13.9–21.1)	14.5 (12.3–17.3)	12.4 (12.2–18.1)	11.1 (9.72–11.9)
KYN (nmol/g ww)	<LOD	<LOD	<LOD	<LOD
KYNA (pmol/g ww)	<LOD	<LOD	<LOD	<LOD
5-HT (pmol/g ww)	4936 (4125–5189)	4302 (4127–4822)	4365 (2312–4520)	4121 (3931–4183)
*Plasma*				
TRP (μM)	28.7 (24.6–32.6)	38.3 (33.9–42.3)	37.9 (36.3–43.0)	31.56 (29.85–39.73)
KYN (μM)	1.37 (0.635–1.72)	1.35 (0.843–1.64)	1.08 (0.578–1.29)	1.16 (0.814–1.39)
KYNA (nM)	41.92 (32.0–58.8)	<LOD∗∗∗^**,###,¥¥¥**^	42.4 (40.7–46.9)	38.8 (36.1–50.6)

5-HT serotonin, CO1 – control group belonging to the cuprizone treated group (DEM) in the demyelination phase; CO2 – control group in the remyelination phase, DEM demyelination group, KYN kynurenine, KYNA kynurenic acid, LOD limit of detection, REM remyelination group, TRP tryptophan, ww wet weight.

∗p < 0.05 *vs*. CO1, ∗∗p < 0.01 *vs*. CO1, ∗∗∗p < 0.001 *vs*. CO1,^#^p < 0.05 *vs*. CO2,^###^p < 0.001 *vs.* CO2, ^¥^p < 0.05 *vs.* REM, ^¥¥¥^p < 0.001 *vs.* REM.

† one animal died in cage after anesthesia.

## 4 Discussion

In the present study, we investigated tryptophan and the metabolites of the KP in the CPZ-induced demyelination model. In this animal model of progressive form of MS, the demyelination occurs in the absence of inflammation, which in turn gives an opportunity to study neurodegenerative processes. Our findings show that CPZ intoxication caused an extensive and severe myelin damage followed by a pronounced astrogliosis at the onset of acute demyelination, thereby reproducing the results previously described in the literature (Praet et al., 2014; Sen et al., 2019) and proving the CPZ model works properly in our case too. Behavioral tests did not show significant differences between experimental groups. These results are also in line with prior data, regarding both rotarod performance, and open field test. However, results on behavioral effects of CPZ are rather controversial (for a detailed review, see (Sen et al., 2019)). In our study, CC was used for IHC measurements, a choice based on other IHC data from literature, item due to the small area of this structure and its unreliable preparation, while for bioanalytical measurements, we selected the brain regions most affected by demyelination and oligodendroglial cell death in the CPZ model (Praet et al., 2014; Sen et al., 2019), but we wanted to expand this spectrum, so we decided to quantify from the remaining brain regions too.

During the examination of the KP metabolites, a significantly decreased KYNA concentration was observed in the CPZ treated DEM group compared to the CO group in the acute demyelination phase, in the cortex and the hippocampus, which difference disappeared during the REM phase, with no significant differences between the samples of the REM group compared to the CO samples. The same pattern was observed in the plasma samples. TRP showed no significant differences in either CNS or blood samples, similarly to the results of Goldberg and his coworkers (Goldberg et al., 2013) in liver function test with no difference in plasma TRP levels between CPZ treated- and control groups. Furthermore, no significant difference was found in KYN and 5-HT levels between the groups during the experiment. This suggests that CPZ may influence KYNA levels directly.

The presumed direct effect of CPZ may derive from the inhibition of KYNA synthesis. Four isoforms of KAT are responsible for the formation of KYNA, with a predominant role of KAT II in mouse liver, and KAT IV in adult mouse brain (Guidetti et al., 2007). The increased concentration of Cu^2+^ inhibits the KAT enzymes, thus it may affect the concentration of KYNA (El-Sewedy et al., 1974). Moreover, CPZ alters the Cu^2+^, Mn^2+^ and Zn^2+^ homeostasis in plasma, kidney, liver and in some brain regions (Moldovan et al., 2015). The explanation seemed to be the decrease in the amount of copper-dependent enzymes (Venturini, 1973) which led to the increase of copper and which further led to the inhibition of the KAT enzyme, and as a consequence to the decrease of KYNA. Although it is not thoroughly studied in the CPZ model, other models of MS have described the expression of KAT enzymes, namely KAT I and KAT II, which were significantly down-regulated in the CNS tissues of the severe experimental autoimmune encephalomyelitis (EAE) mice, accordingly decreased KYNA levels were observed in the tissues (Sundaram et al., 2020). Furthermore, this was simultaneously supported by increased kynurenine 3-monooxygenase (KMO) enzyme expression in the CNS tissues examined, that is the KYNA/QUIN ratio was significantly decreased in the EAE mice compared to the control group (Sundaram et al., 2020).

On the other hand, CPZ may alter the KYNA synthesis via damaging astrocytes. During intoxication, damaged astrocytes appear, with morphological change and become hypertrophic (Praet et al., 2014), with an extensive astrogliosis. We can assume that this astrocyte activation also alters KYNA synthesis.

It is also possible that the KP is shifted towards neurotoxic metabolites and this is responsible for the decrease in KYNA concentration. The CPZ model is an rodent model of progressive form of MS to investigate the demyelination and remyelination without immune response (Sen et al., 2019). In our study, we found a decreased KYNA concentration in the CPZ-treated DEM group during demyelination, however, in human MS studies, elevated KYNA levels have been reported during active relapse phase, and decreased during remission, as well as in progressive forms of the disease (Lim et al., 2017; Lovelace et al., 2016; Rajda et al., 2015; Vécsei et al., 2013). This observation raises the possibility, that the KP has a complex role in the pathomechanism of MS, it behaves differently in short and long term, and it responds differently in immune-mediated and non immune-mediated conditions, as MS is an immune-mediated disease of CNS, in which the KP, including the IDO enzyme, is greatly affected by the immune pathways (Vécsei et al., 2013). Nevertheless, the CPZ-induced demyelination model is a primary oligodendropathy model (Praet et al., 2014), and although it cannot induce inflammation, it affects the KP.

In the CPZ-induced model, microglia and macrophage activation occur in the early stages of demyelination (Praet et al., 2014). Furthermore, myelin debris has a regulatory function for microglia activation, phagocytosis and consequently the process of remyelination (Kotter et al., 2006; Skripuletz et al., 2013). Within CPZ-induced demyelinated lesions, both pro-, and anti-inflammatory phenotypes can be attributed to microglia (for a detailed review, see (Praet et al., 2014). In a previous study, macrophage/microglia cells were described as the major QUIN expressors (Guillemin et al., 2003), suggesting that a prolonged inflammatory response may elicit an elevation in QUIN concentration in a pathophysiological range (Sundaram et al., 2020). Moreover, it has been reported, that QUIN produced by activated monocyte cells, in pathophysiological concentration can cause neuronal and glial cell death (Guillemin et al., 2001), and these cells are capable of inducing demyelination by direct association with oligodendrocytes (Yamasaki et al., 2014).

In the light of the above provided literature information, in the CPZ-induced demyelination model, KP may shift in a neurotoxic direction, which may be responsible for the drastic decrease of the KYNA level.

The CPZ-induced mitochondrial dysfunction, consequent energy deficit and increased oxidative stress may initiate a cascade, which results in demyelination and neurodegeneration (Praet et al., 2014). In this concept, CPZ may not inhibit the synthesis, merely KYNA is consumed in larger quantities than it is formed, due to its ROS scavenger effect (Vécsei et al., 2013). Because of its involvement in the above mentioned potentially neurotoxic processes, KYNA is considered a neuroprotective agent (Bohár et al., 2015). Consequently, due to the fact, that in our study the levels of KYNA decreased both in plasma and CNS, we assume, that its protective property is less pronounced, whereas the neurodegeneration is way more enhanced, thus the excitotoxicity may be more significant.

**Figure 11:**
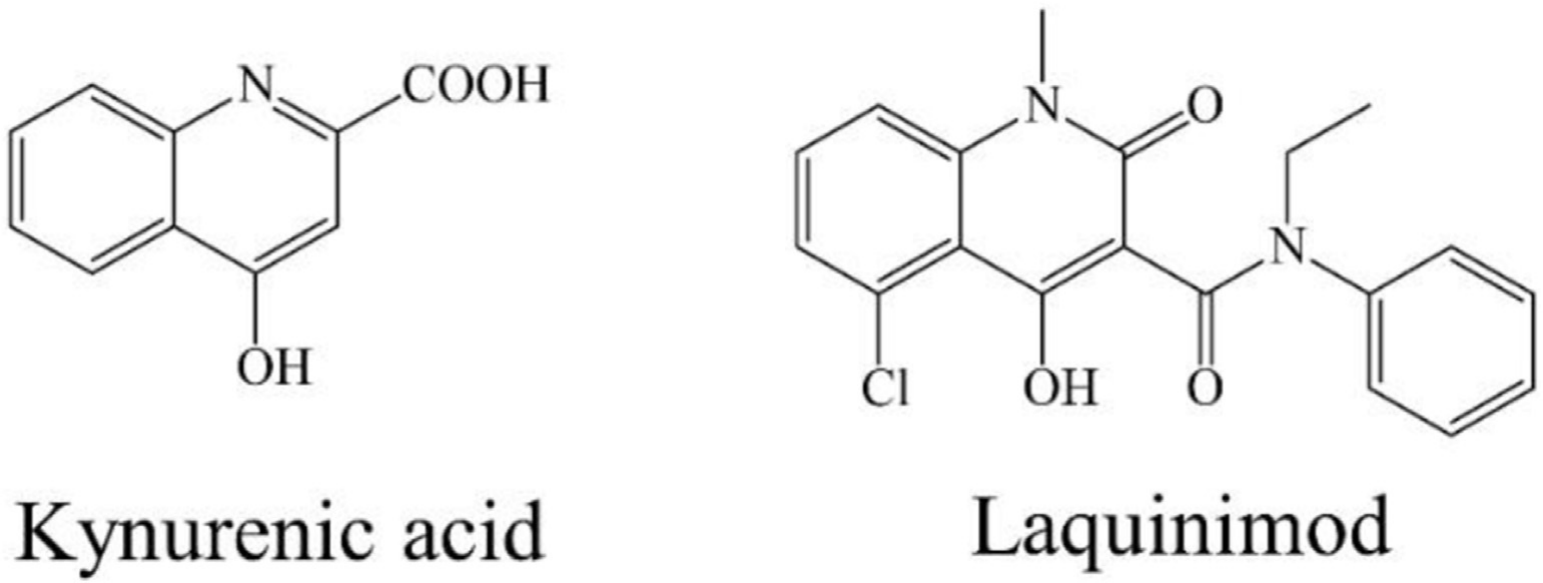
Structural formula of KYNA and laquinimod

In a recent study (Nedelcu et al., 2020) the well-known small molecule laquinimod ‒ showing structural similarities with KYNA (Figure 11) ‒ was described to be capable to ameliorate inflammatory demyelination, metabolic OLG injury, with further anti-inflammatory effects in the CPZ model. Furthermore, it has potential benefits in clinical cases by suppressing the development of active lesions in relapsing-remitting MS (Comi et al., 2008; Polman et al., 2005). In addition, laquinimod is able to reduce the degree of axonal damage and demyelination, as well as inflammatory lesions in the EAE model of MS (Aharoni et al., 2012; Brück et al., 2012). Moreover, laquinimod influences the glutamatergic and GABAergic transmission in the striatum of EAE mice (Ruffini et al., 2012). In a recent *in vitro* study, it was proved, that laquinimod improved cerebral glutamatergic transmission on cerebellar slices of EAE mice (Gentile et al., 2018). It seems, that the exogenously administered KYNA analogue is able to reduce the degree of damage therefore it is assumed that the KYNA is consumed during demyelination.

The limits of our study are the relatively few measured KP metabolites, and the lack of cognitive function tests during behavioral investigation.

Nevertheless, this is the first study assessing some of the TRP metabolites changes in five different brain regions and plasma after CPZ treatment, confirming decreased KYNA levels in both sides of the blood-brain barrier during demyelination. These findings support the involvement of the KP in the pathogenesis of MS. Although the above-described theories serve as an initial hypothesis, it is clear that further studies are needed to be conducted to determine, what is the exact mechanism of the reduction in KYNA level during CPZ treatment and how this phenomenon may be related to de- and remyelinating processes and the possible effects of the KP on demyelination of the CNS. Our future plan is a detailed exploration of the KP in the CPZ intoxicated mouse model, besides on the use of specific agonists and antagonists of the KP.

## Declarations

### Author contribution statement

Helga Polyák, Edina Katalin Cseh: Performed the experiments; Analyzed and interpreted the data; Wrote the paper.

Zsuzsanna Bohár: Analyzed and interpreted the data; Contributed reagents, materials, analysis tools or data; Wrote the paper.

Cecilia Rajda, Dénes Zádori, József Toldi: Contributed reagents, materials, analysis tools or data; Wrote the paper.

Péter Klivényi, László Vécsei: Conceived and designed the experiments; Contributed reagents, materials, analysis tools or data; Wrote the paper.

### Funding statement

This work was supported by GINOP-2.3.2-15-2016-00034, EFOP-3.6.1-16-2016-00008, 20391-3/2018/FEKUSTRAT Ministry of Human Capacities, Hungary and MTA-SZTE Neuroscience Research Group and by University of Szeged Open Access Fund, Grant number: 5021. Helga Polyák was supported by the ÚNKP-20-3 - New National Excellence Program of the Ministry for Innovation and Technology from the source of the National Research, Development and Innovation Fund; and EFOP 3.6.3-VEKOP-16-2017-00009. Edina Katalin Cseh was supported by UNKP-19-3 New National Excellence Program of the Ministry for Innovation and Technology and EFOP-3.6.3-VEKOP-16-2017-00009.

### Data availability statement

Data will be made available on request.

### Declaration of interests statement

The authors declare no conflict of interest.

### Additional information

No additional information is available for this paper.
